# Survival Analysis by Penalized Regression and Matrix Factorization

**DOI:** 10.1155/2013/632030

**Published:** 2013-04-23

**Authors:** Yeuntyng Lai, Morihiro Hayashida, Tatsuya Akutsu

**Affiliations:** Bioinformatics Center, Institute for Chemical Research, Kyoto University, Gokasho, Uji, Kyoto 611-0011, Japan

## Abstract

Because every disease has its unique survival pattern, it is necessary to find a suitable model to simulate followups. DNA microarray is a useful technique to detect thousands of gene expressions at one time and is usually employed to classify different types of cancer. We propose combination methods of penalized regression models and nonnegative matrix factorization (NMF) for predicting survival. We tried *L*_1_- (lasso), *L*_2_- (ridge), and *L*_1_-*L*_2_ combined (elastic net) penalized regression for diffuse large B-cell lymphoma (DLBCL) patients' microarray data and found that *L*_1_-*L*_2_ combined method predicts survival best with the smallest logrank *P* value. Furthermore, 80% of selected genes have been reported to correlate with carcinogenesis or lymphoma. Through NMF we found that DLBCL patients can be divided into 4 groups clearly, and it implies that DLBCL may have 4 subtypes which have a little different survival patterns. Next we excluded some patients who were indicated hard to classify in NMF and executed three penalized regression models again. We found that the performance of survival prediction has been improved with lower logrank *P* values. Therefore, we conclude that after preselection of patients by NMF, penalized regression models can predict DLBCL patients' survival successfully.

## 1. Introduction

Survival analysis is a branch of statistics that is of interest to researchers in when patients' death will occur after some therapies [1]. So far there are many methods to analyze survival data, for example, Kaplan-Meier curve, logrank test, Cox proportional hazards model, and so on. We often have information about patients' survival status and survival time. However, censored data cannot offer complete information; that is to say, the survival time of live patient is only partially known. Because of such censored data, survival analysis becomes more complicated than other studies.

The Kaplan-Meier curve is the most popular illustration of survival pattern, and it only considers the survival time data of dead patients (excluding the censored data). By Kaplan-Meier curve, we can estimate the survival rate at different survival time. The logrank test is a useful method to compare the survival distributions, where we can consider the logrank test as a modified chi-squared test. The Cox proportional hazards model is the most famous regression model in survival analysis. Its main concept is to analyze the relationships between multiple covariates and survival time. The covariates may be internal factors such as patients' age, sex, or gene expression, whereas external factors may include environmental influences like smoke, food, or life style. Since survival time is most likely not normally distributed, we cannot directly use original multiple regression to simulate regression models. The survival patterns usually display as exponential or Weibull distributions. In addition, the survival data have the “censored” problem; therefore, we need a special regression method, like Cox regression model, to perform survival analysis. We will discuss it in detail in Section 2.

So far there is some research in linking gene expression profiles to survival data, such as predictions of therapy outcome in kidney [2], lung [3], and breast cancer [4]. The traditional procedures are utilizing Cox regression model to select significant genes [5] or separating patients into different risk levels by hierarchical clustering [2]. Because of high dimension of microarray data, some researchers introduce partial least squares [6] or least angle regression [7] to reduce the dimension. An optimized set of guidelines has been published to utilize penalized regression dealing with gene expression data [8]. Sparse kernel methods also have been employed as survival SVM and IVM and could get better results than Cox regression [9]. Some researchers apply Bayesian approach to add flexibility accounting for nonlinear relationships between survival time and gene expression level [10]. Unlike focusing on the problem of high dimension within microarray data, selecting patients whose survival patterns are extremely different also can improve survival prediction performance [11]. Here we are trying to use microarray data to predict survival by combining two kinds of methods: (1) penalized regression models and (2) nonnegative matrix factorization.

Furthermore, we choose the disease, diffuse large B-cell lymphoma (DLBCL) to analyze, because this disease has diagnostic discrepancies if only based on clinical morphology [12]. DLBCL is the most common subtype of non-Hodgkin's lymphoma and accounts for approximately 40% in adults. The DLBCL patients can be cured by chemotherapy with only 35 to 40 percent. The dataset [13] can be downloaded from http://llmpp.nih.gov/DLBCL. It contains a total of 240 patients with untreated DLBCL, and all of the patients have no previous history of lymphoma. The median followup is 2.8 years for total patients and 7.3 years for survived patients. During this study 138 patients (57%) unfortunately died. The tumor samples of DLBCL patients are collected and tested by DNA microarray experiment. The cDNA clones on the Lymphochip microarray are composed of genes that are considered to express in lymphoid cells and some genes that are thought or confirmed to play a role in cancer or immune function. Each microarray datum of each patient comprises 7395 different genes, but some genes of some patients have too weak fluorescent signals (compared with dot's surrounding) and are denoted as missing values. There are only 434 genes without missing values among total 240 patients. The Cy5/Cy3 ratios are log transformed by base 2 and stored in a table to construct gene expression profiles.

## 2. Methods

### 2.1. Cox Proportional Hazards Model

 The Cox proportional hazards model is constructed by Cox [14] and widely used in the analysis of survival data. The Cox regression model demonstrates that the hazard function *h*(*t*), which means the risk of death at time *t* for an individual with gene expression profiles, is given by

(1) $$\begin{array}{c} h\left(t\;|\;X\right)=h_{0}\left(t\right)\exp\left(\sum_{i=1}^{p}\beta_{i}x_{i}\right)=h_{0}\left(t\right)\exp\left({}^{⊤}\beta X\right), \end{array}$$

where *h*_0_(*t*) is the baseline hazard, **β** = ^*⊤*^(*β*_1_,…, *β*_*p*_) is the column vector of regression parameters, and ^*⊤*^ **β** means its transpose. **X** = ^*⊤*^(*x*_1_,…, *x*_*p*_) denotes the gene expression levels of *p* genes. The term *h*_0_(*t*) is the hazard when all gene expression levels are equal to zero. Or we can think the Cox proportional hazards model as another form:

(2) $$\begin{array}{c} \log\frac{h\left(t\;|\;X\right)}{h_{0}\left(t\right)}={}^{⊤}\beta X. \end{array}$$

In Cox regression model, there is no assumption about the probability distribution of the hazard. It just assumes that the ratio of hazard functions of different observations does not depend on time [1]. The other assumption is that there is a log-linear relationship between covariates (gene expression levels) and hazard function. Finally we can presume the Cox proportional hazards model as a modified “simple” linear regression model. Like other statistical methods using likelihood function to estimate parameters from a dataset, in Cox proportional hazards model, the Cox partial likelihood is also derived by Cox [14] as follows:

(3) $$\begin{array}{c} L\left(\beta \right)=\prod_{r\in D}\frac{\exp\left({}^{⊤}\beta X^{\left(r\right)}\right)}{\sum_{j\in R_{r}}\exp\left({}^{⊤}\beta X^{\left(j\right)}\right)\;}, \end{array}$$

where *D* is the set of indices of patient death and *R*_*r*_ denotes the set of indices of the individuals at risk for death at time *t*_*r*_ [7]. For many applications of likelihood function, the term that takes logarithm of likelihood is more convenient than the original likelihood function. Thus, taking the logarithm of the Cox partial likelihood, we have the following log partial likelihood:

(4) $$\begin{array}{c} l\left(\beta \right)=\sum_{r\in D}\left({}^{⊤}\beta X^{\left(r\right)}-\log\left(\sum_{j\in R_{r}}\exp\left({}^{⊤}\beta X^{\left(j\right)}\right)\right)\right). \end{array}$$

Next we follow the normal maximum likelihood estimation method to calculate unknown parameters. Our goal is to estimate the regression coefficients **β**, so we can maximize the log partial likelihood function over **β**.

### 2.2. Lasso, Ridge, and Other Penalized Regression

 In usual cases that the patient size *n* is bigger than covariate number *p*, we can compute **β** by maximizing the log partial likelihood. However, some research has indicated that the Cox proportional hazards model cannot be applied directly to predict survival time when *p* ≫ *n* (e.g., in microarray case) [7, 15]. It is because of the high-dimensional space of the predictors and high collinearity of some genes. When we use microarray data to do survival analysis, the dataset always composes thousands of gene expression data. The huge gene numbers make the prediction model a very high dimensional and cause the difficulty of computing. The second problem usually happens in biological research, because the expression levels of some genes are highly correlated. These genes may belong to the same biological pathway or play similar roles in different reactions. To solve these problems, we apply several kinds of penalized regression methods.

All of the penalized regression models are based on the Cox proportional hazards model. The idea is to add a regularization term in the Cox partial likelihood function and control the over-fitting. There are two popular kinds of penalized regression methods. The first one is *L*_1_-penalized regression; it is also called the least absolute shrinkage and selection operator (lasso) estimation, which was first proposed by Tibshirani [16]. Because of some constraints in lasso's principles, it tends to convert some coefficients to zero finally. According to this special characteristic, the lasso estimation is often applied in parameter shrinkage to build simpler models. The second penalized regression method is *L*_2_-penalized regression, which is usually called ridge regression [17]. Unlike lasso estimation, ridge regression conserves all parameters to construct prediction models.

To add regularization term into the Cox regression model, the log partial likelihood function will be rewritten as the following. *L*_1_-penalized (lasso) log partial likelihood is given by

(5) $$\begin{array}{c} l\left(\beta \right)-\sum_{j=1}^{p}\lambda \left|\beta_{j}\right|, \end{array}$$

and *L*_2_-penalized (ridge) log partial likelihood is written as

(6) $$\begin{array}{c} l\left(\beta \right)-\sum_{j=1}^{p}\lambda \beta_{j}^{2}, \end{array}$$

where *λ* is a tuning parameter and *p* is the number of genes. There is another simple penalized likelihood method combined with *L*_1_- and *L*_2_-penalized regression. It is named the elastic net, and its log partial likelihood is

(7) $$\begin{array}{c} l\left(\beta \right)-\sum_{j=1}^{p}\left(\lambda_{1}\left|\beta_{j}\right|+\lambda_{2}\beta_{j}^{2}\right), \end{array}$$

where *λ*_1_ and *λ*_2_ are corresponding tuning parameters of *L*_1_ and *L*_2_ penalties, respectively. We can find that the elastic net method just adds *L*_1_ and *L*_2_ penalties together to create a new regularization term. The elastic net performs feature selection and parameter estimation as the lasso regression. However, by adding the *L*_2_ penalty it distributes weight to more variables; hence, the elastic net may select more parameters than the lasso regression [18].

In order to select variables, we first randomly divided 240 patients into training group (160 patients) and testing group (80 patients). Although the whole microarray datum contains 7395 genes for each patient, we primarily used the 434 complete genes (without missing values among all patients) to build penalized regression models. All experiments were executed on R platform by using libraries of “survival” and “penalized”. We initially established *L*_1_-, *L*_2_-, and *L*_1_-*L*_2_ combined penalized regression models by training data. No doubt it is necessary to set the tuning parameter *λ* first, and we can use the cross-validation method encompassed in the “penalized” package to find optimal values. If we set tuning parameters too small, the algorithm may run very slowly and fail to converge especially for high-dimensional data. In this study, we set *λ* = 10 in lasso regression model and *λ* = 20 in ridge regression model. In *L*_1_-*L*_2_ combined penalized regression model, we set *λ*_1_ = 10 and *λ*_2_ = 20. After computing on R platform, we got out 21 nonzero coefficients in lasso regression model and 27 ones in elastic net model. In other words, we selected out 21 and 27 important genes.

### 2.3. Nonnegative Matrix Factorization

 The nonnegative matrix factorization (NMF) method has been introduced first to decompose images, and its goal is to factorize a matrix into two nonnegative matrices [19]. In NMF, it makes the constraint about nonnegativity of matrices. It is because not only most data in the real world are nonnegative but also we can only explain their meanings in nonnegative way [20]. The other characteristic of NMF is the additive property; that is to say, the NMF model does not allow subtraction. This special signature makes NMF illustrate quantitatively each component. Or we can say NMF is a part-based representation method. While zero value represents the absence of some components or events, the positive value may denote the presence of the same ones.

Lee and Seung first used NMF to do image decomposition [21]. They analyzed a face figure with NMF and compared the result with principal component analysis (PCA) and vector quantization (VQ). They showed that NMF can do part-based representation, whereas PCA and VQ represented the face image holistically. In other words, NMF decomposed the face image successfully into several facial parts, like nose, lips, and eyes. Suppose we have the image data matrix *V* of size *n* × *m* that contains *m* facial images, where each image has *n* nonnegative pixels. Our goal is to factorize the matrix *V* into two nonnegative matrices, *W* and *H*:

(8) $$\begin{array}{c} V\approx WH. \end{array}$$

The sizes of the matrices *W* and *H* are *n* × *k* and *k* × *m*, respectively. The rank *k* is usually chosen to be smaller than *n* and *m*, so that *W* and *H* are smaller than the original matrix *V*. The rank *k* is similar to the basis image that identifies parts of the face image.

The NMF method starts by randomly initializing nonnegative matrices *W* and *H*. Similar the values of *V* and *WH* are, the distance between *V* and *WH* approaches approximately to zero. A useful distance measurement is to calculate the square of the Euclidean distance between them. The equation can be written as

(9) $$\begin{array}{c} {||V-WH||}^{2}=\sum_{ij}{\left(V_{ij}-{\left(WH\right)}_{ij}\right)}^{2}. \end{array}$$

Another similarity measurement is to test the “divergence” of *V* from *WH*, which is denoted as a divergence function:

(10) $$\begin{array}{c} D\left(V||WH\right)=\sum_{ij}\left(V_{ij}\log\frac{V_{ij}}{{\left(WH\right)}_{ij}}-V_{ij}+{\left(WH\right)}_{ij}\right). \end{array}$$

Unlike the Euclidean distance, it assumes that *V* and *WH* are not symmetric. To minimize the Euclidean distance or the divergence function, Lee and Seung created the following “multiplicative update rules” to ease implementation and accelerate computing speed [21]. The Euclidean distance ||*V* − *WH*|| is nonincreasing under the update rules:

(11) Hau⟵Hau(WV⊤)au(WWH⊤)au,Wia⟵Wia(V⊤H)ia(WH⊤H)ia.

Furthermore, the divergence *D*(*V*||*WH*) is nonincreasing under the update rules:

(12) $$\begin{array}{c} H_{au}⟵H_{au}\frac{\sum_{i}W_{ia}V_{iu}/{\left(WH\right)}_{iu}}{\sum_{k}W_{ka}},\\ W_{ia}⟵W_{ia}\frac{\sum_{u}H_{au}V_{iu}/{\left(WH\right)}_{iu}}{\sum_{v}H_{av}}. \end{array}$$

Therefore, we can iteratively update *W* and *H* to minimize the Euclidean distance or the divergence function by upper coupled update rules.

It has been indicated that NMF is very useful when analyzing data that have multiple attributes, and these attributes are often ambiguous and hard to predict. Because of this property, NMF has been applied much in text data mining. The same word may have other different meanings just depending on the different locations in the sentence or document. It resembles the biological data so that the same gene may play different roles in different biological pathways. To deal with the gene complexity of multiple functions, NMF method has been exploited to process the biomedical data such as microarray data [22].

In microarray case, we first consider the gene expression matrix *A*, which is composed by *N* genes in *M* patients. In other words, the size of matrix *A* is *N* × *M*. Then, we want to factorize matrix *A* into two matrices with nonnegative entries, *A* ≈ *WH*. It means to find a small number of genes (which are called metagenes) to represent the whole gene expression pattern of patients [23]. That is to say, we can approximate the gene expression pattern as positive linear combinations of these metagenes. Like to find the essential face components (eyes, nose, and lips) from the entire face image, we try to figure out the representative metagenes that may provide biological insight into sparse microarray data. Each column of *W* of size *N* × *k* defines a metagene and each column of *H* of size *k* × *M* defines the metagene expression pattern of the corresponding patient, where the *ij*th elements *w*_*ij*_ and *h*_*ij*_ represent the coefficient of gene *i* in metagene *j* and the expression level of metagene *i* in patient *j*, respectively.

Figure S1 (see Supplementary Material available online at http://dx.doi.org/10.1155/2013/632030) shows a simple example when rank *k* = 2. We can see the original gene expression matrix *A* is decomposed into two smaller matrices *W* and *H*. There are two ways to analyze the gene expression pattern—the matrix *W*-based aspect and the matrix *H*-based aspect. In *W*-based view, the total *N* genes can be grouped into some clusters according to the value of entries *w*_*ij*_. If we reorder the *N* genes by the coefficients of every gene in corresponding metagene, the inherent special expression pattern of some genes may be uncovered. In *H*-based view, the *M* patients can be clustered into *k* groups by the expression levels of metagene for all patients. As Figure S1 shows, metagene expression profiles are illustrated in two significant distributions (red line and blue line). By this unique distribution, we can cluster patients into cluster 1 and cluster 2 separately. Because of this distinctive character, nonnegative matrix factorization can be employed as a clustering method in microarray data. Moreover by reason of the dual-way aspect of *W* and *H* matrices, some research has proved it is practicable to analyze the microarray data in the biclustering way [24].

Since we can set any rank *k* to group patients into *k* clusters, the key point is to find *k* that can partition patients into meaningful clusters. To solve this problem, we apply the method of consensus clustering [23]. The different initial matrices of *W* and *H* on each run may cause different clustering forms of patients. However, if rank *k* is strong enough, we may expect that patient assignment to clusters would vary a little from run to run. For each run, the patient assignment can be defined by a connectivity matrix *C*. The size of matrix *C* is *M* × *M*, and entry *c*_*ij*_ = 1 if patient *i* and patient *j* belong to the same cluster. Whereas entry *c*_*ij*_ = 0 if patient *i* and patient *j* belong to different clusters. Then, we compute the average connectivity matrix *C* over 100 runs and denote it as the consensus matrix $\overset{-}{C}$. Since *c*_12_ takes 1 or 0 over 100 runs as shown in Figure S2, the average of *c*_12_ will be within the region between 1 and 0. Therefore, all the entries of $\overset{-}{C}$ may range from 0 to 1 and reflect the probability that patient *i* and patient *j* are assigned to the same cluster. Next, we can reorder all patients by their assignment probability and then construct a new consensus clustering matrix by heat map presentation coloring from 0 (deep blue, patients in different groups) to 1 (dark red, patients in the same group). Through heat map result, we can evaluate the validity of any setting rank *k*. All experiments were executed on R platform by using the library of “NMF”.

The gene expression levels in microarray are displayed as Cy5/Cy3 log-2 ratios, and these values are distributed dispersedly as positives or negatives. Additionally, there are only 434 genes without missing values among total 240 patients. Since the missing values are caused by the too weak fluorescent signals to detect, we may think these values are approximately equal to zero. So, we refilled all the missing values in the gene expression profiles as zero [25]. Next procedure is to transform all of the ratios into nonnegative values; therefore, we used each ratio as an exponent by base 2 [22].

### 2.4. Lasso Regression after NMF Selection

 According to the consensus clustering results by NMF, we found some patients cannot be clustered into the same group over all 100 runs. We may suggest that these patients will become noise in the following computing. Therefore, we excluded the patients whose value in $\overset{-}{C}$ is smaller than 0.9. We finally excluded 15 patients from the training group and 7 patients from the testing group. Next, we built *L*_1_-, *L*_2_-, and *L*_1_-*L*_2_ combined penalized regression models again.

To compare the prediction performance of the three penalized regression models, we should define the criteria of prediction assessment initially. However, there are no determinate criteria that have been stipulated for survival analysis [26]. Furthermore, many comparative studies of survival prediction have indicated that different criteria may influence the conclusion about evaluations of different prediction models [15, 27]. We chose one simple evaluation criteria that has been reported in many survival studies. A common way to assess the effect of one prediction model is to check whether or not the assignments of patients, such as “high-risk” group or “low-risk” group, are correct. In clinic, patients are always concerned about whether or not they are at risk for death after some therapies.

Let ${\hat{\beta }}_{\text{train}}$ denote the vector of estimated regression coefficients obtained from training data. For each patient *i* in the testing group, this estimate is then used together with its vector of gene expression values **X** ^(*i*)^ to derive a prognostic index *η*_*i*_ for the patient, given by $\eta_{i}={}^{⊤}{\hat{\beta }}_{\text{train}}X^{\left(i\right)}$ [27]. Then, we found the median of the prognostic indices of 80 patients. If the prognostic index is bigger than the median, the patient is assigned to the high-risk group, whereas smaller than the median the patient is assigned to the low-risk group. We can compare the results of *L*_1_- (lasso), *L*_2_- (ridge), and *L*_1_-*L*_2_ combined (elastic net) penalized regression model by the Kaplan-Meier curve.

## 3. Results

### 3.1. Important Genes Selected out by Lasso Regression

We have described in Section 2 that there are 21 genes selected out by lasso regression model and 27 genes selected out by elastic net model. Moreover, the 21 genes are overall included in the 27 genes. It implies that these 21 genes (see Table 1) may play important roles in patients' survival. To understand these genes more comprehensively, we investigated their biological functions and discriminated whether or not they are involved in carcinogenesis. We found that there are 10 genes that have been reported to relate to some cancers, and 5 genes of them are indicated to influence the DLBCL patients' survival [13]. Two genes are tumor suppressor genes or oncogenes and hint them playing noticeable roles in carcinogenesis. On the other hand, there are 9 genes that have biological functions concerned to fundamental immune functions, such as MHC class II or antigen processing. We may infer that genes with these special biological functions will cause DLBCL pathogenesis and even affect patients' survival eventually. Unfortunately, the biological functions of two genes within the 21 genes have not been known clearly so far. However, total 17 genes among the 21 genes (about 80 percent) are correlated to carcinogenesis or important immune functions. It makes us believe that the *L*_1_- and *L*_1_-*L*_2_ combined penalized regression models may select out significant genes associated to the DLBCL patients' survival.

### 3.2. Divide DLBCL into 4 Subgroups by NMF

We initially used 434 gene expression profiles (without missing values) to run matrix factorization 100 times for rank *k* = 2,…, 5 and got the consensus matrix. The reordered consensus clustering results are illustrated by heat maps in Figure S3. We found that the clustering pattern is better when rank *k* = 3 or 4 and is the worst when rank *k* = 5. It suggests that 3 or 4 groupings of DLBCL patients may have some biological meaning, so we next plotted the Kaplan-Meier curve of two-divided, three-divided, and four-divided results as Figure S4 shows to compare their survival distributions. Using logrank test, we also calculated the *P* value of each result and got 0.927, 0.13, and 0.00365 from rank *k* = 2 to 4. We found that only when rank *k* = 4 the survival curves separate significantly among four patient groups (the *P* value is smaller than 0.05), meaning that the fourth division of DLBCL patients has some biological implications that may generate different survival patterns of patients.

**Figure 1:**
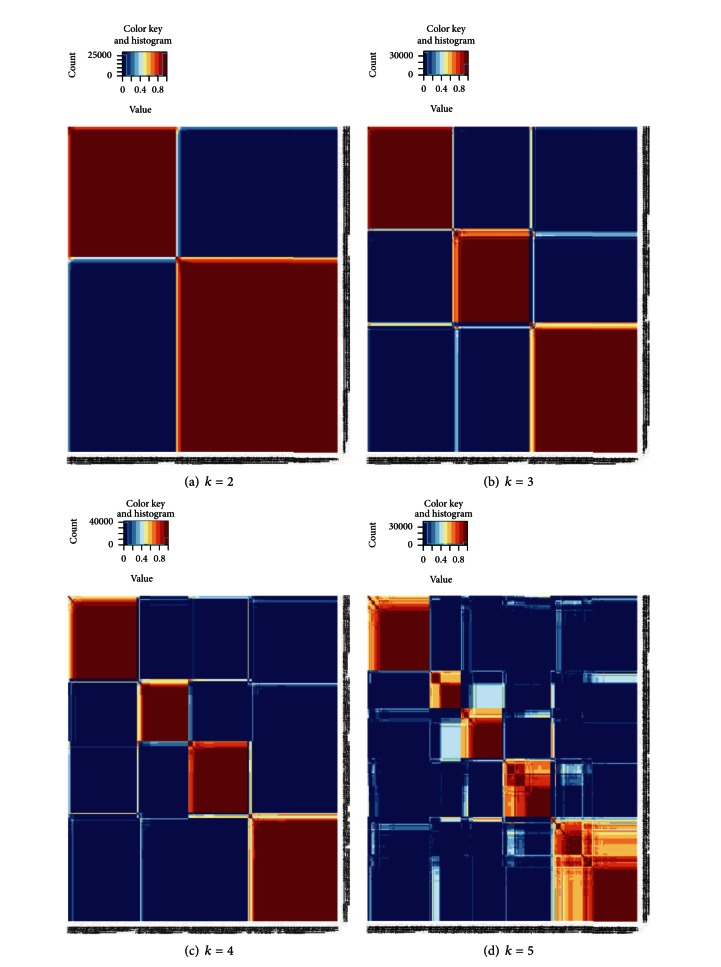
Consensus clustering results of 7395 genes for rank *k* = 2,…, 5.

**Figure 2:**
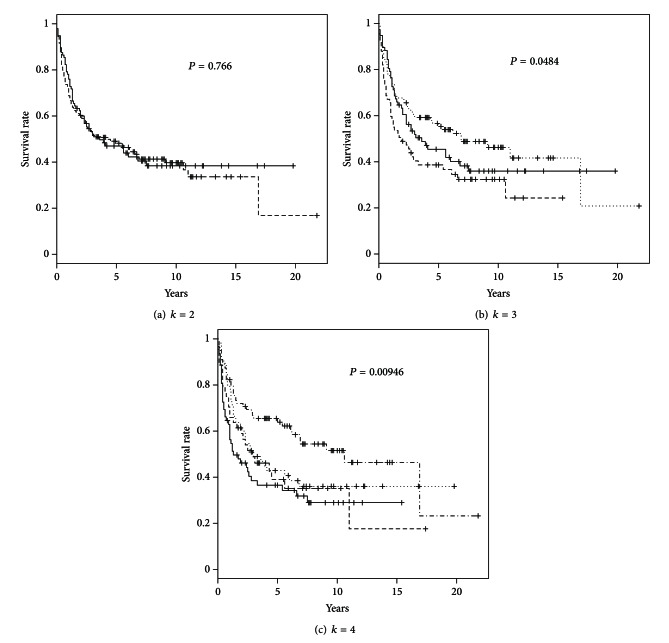
The Kaplan-Meier curve results of three kinds of patients' divisions corresponding to rank *k* = 2,…, 4.

Since the survival curves did not separate significantly in two-division and three-division results, we changed to use all of the 7395 genes (missing values approximated to zero) to analyze again. Similarly after running 100 nonnegative matrix factorizations, the heat maps of the consensus matrix for rank *k* = 2,…, 5 are shown in Figure 1. We found that the clustering pattern is good when rank *k* = 2,3, or 4 and is the worst when rank *k* = 5. However, comparing with the results of 434 genes generally, all clustering results of 7395 genes are much better. Next, we plotted likewise the Kaplan-Meier curve of two-divided, three-divided, and four-divided results in Figure 2 and compared their survival distributions. By using logrank test again, we measured each *P* value of survival curves, which yielded 0.766, 0.0484, and 0.00946 from rank *k* = 2 to 4. We discovered that the *P* values of not only rank *k* = 4 but also *k* = 3 are smaller than 0.05. It implies that the survival patterns can be distinguished significantly when DLBCL patients are divided into 3 or 4 groups.

### 3.3. Survival Prediction of Lasso Model is Improved by Preselection of NMF

**Figure 3:**
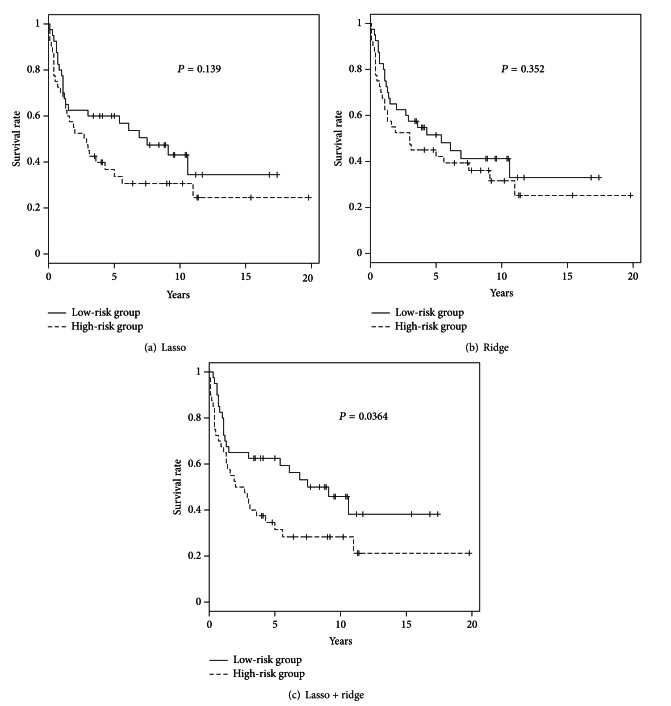
The Kaplan-Meier curve results of three penalized regression models, lasso, ridge, and elastic net, for low- and high- risk groups, respectively.

We compared the survival predictions of *L*_1_- (lasso), *L*_2_- (ridge), and *L*_1_-*L*_2_ combined (elastic net) penalized regression models by the Kaplan-Meier curve as showing in Figure 3. Using logrank test, we also calculated the *P* value of each model and got 0.139, 0.352, and 0.0364 from top to bottom. In all three models, the patients' survival rates of low-risk group are always higher than the survival rates of high-risk group. We found that only in *L*_1_-*L*_2_ combined penalized regression model, the survival curves separate significantly between high-risk group and low-risk group (the *P* value is smaller than 0.05), meaning that the elastic net model successfully predicts followups with high risk or low risk of death.

**Figure 4:**
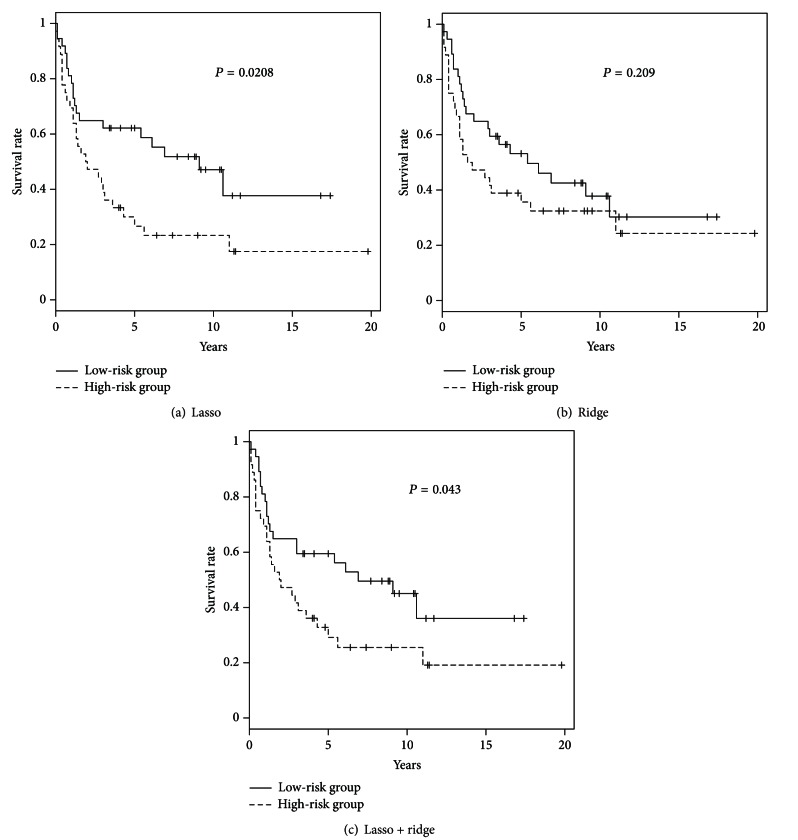
The Kaplan-Meier curve results of three penalized regression models, lasso, ridge, and elastic net, for low- and high- risk groups, respectively, after preselection of NMF.

After exclusion of noise patients by NMF, we tested *L*_1_-, *L*_2_-, and *L*_1_-*L*_2_ combined penalized regression models again. Similarly plotting the Kaplan-Meier curve in Figure 4, we yielded the *P* values as 0.0208, 0.209, and 0.043 by logrank test. The *P* values of *L*_1_- and *L*_2_- penalized regression models became smaller, whereas bigger in *L*_1_-*L*_2_ combined model. However, the survival distributions in *L*_1_-*L*_2_ combined penalized regression model are still significant between high-risk and low-risk groups. Consequently, we may conclude that the prediction performance can be improved (especially in lasso regression model) by previously excluding some patients who are considered hard to classify in NMF.

## 4. Discussion

Through three penalized regression methods based on Cox proportional hazards model, we analyzed the microarray data of DLBCL patients and tried to predict the patients' survival. We found that without preselection of NMF, *L*_1_-*L*_2_ combined (elastic net) penalized regression model yields better prediction performance than *L*_1_- (lasso) and *L*_2_- (ridge) penalized regression models because of the smallest *P* value. It seems that the elastic net method combines the advantages of both lasso and ridge regression methods. Furthermore, the elastic net method reserves the merit of lasso regression that can sort out the important genes that may influence the patients' survival. To improve the prediction performance of *L*_1_-*L*_2_ combined penalized regression model, different kinds of combination or modification should be developed.

21 genes were selected out by *L*_1_- and *L*_1_-*L*_2_ combined penalized regression models. Among them, MYC, HLA-DQA1, HLA-DPA1, HLA-DRB1, and CD22 have been reported to be used in prediction of DLBCL patients' survival [13]. Moreover, MYC has been indicated as an oncogenic transcription factor that regulates expressions of a great number of genes. CD22 is a B-cell marker and regulates the signaling pathways within B cell. Recent research shows that CD22 is a potential drug target in many cancers.

In this study, we only utilized gene expression data as predictors. However, prediction performance may be improved by adding other covariates such as age, sex, and stage [5]. Unfortunately, the DLBCL dataset does not contain detailed information about clinical data. Nevertheless not only clinical factors but also published gene signatures that are employed in some cancer prediction chips are proved to increase the predictive strength [28].

We employed the nonnegative matrix factorization to naturally cluster DLBCL patients into some groups and then compared the survival distributions within different groupings. Not only complete gene expression profiles but also total gene expression profiles indicate that the patient grouping of 4 has some biological meaning. It implies that the disease DLBCL may have 4 subtypes that have a little different survival patterns. Moreover, if we observe the heat maps of consensus clustering matrix more carefully, we will find some patients with values near to 1 but not equal to 1 (the orange or yellow color). It means these patients do not always belong to the same group and may suggest that they have unusual gene expression profiles because of special constitutions or other unknown diseases.

The R package of “NMF” provides other distance calculation methods to execute, such as nsNMF, offset, pe-NMF and snmf. Examining these different algorithms may get different consensus clustering results. Another useful NMF algorithm which is called Semi-NMF can handle clustering while input data contain negative values. Its plug-in for a microarray data analysis tool has even been introduced [25]. Of course, NMF can deal with other kinds of data different from microarray data. Array comparative genomic hybridization data are also utilized to analyze patients' survival [29].

The DLBCL dataset that we used in this study has been analyzed by hierarchical clustering before [12, 13]. Although they claim to cluster DLBCL patients into 2 or 3 groups, our NMF results prefer 4 groups. It may be because of distinct algorithms within two methods. Nevertheless, the consistent results are also reported in lung cancer case [22].

An obvious problem in microarray data is the existence of missing values. To make full use of gene expression profiles, we should employ some methods to estimate missing values. For example, a nearest neighbor technique has been employed to approximate missing values in DLBCL microarray dataset and then predict patients' survival well [7, 30].

There is a growing tendency in research about survival analysis for the last several decades. Many new ideas from different fields have been introduced to predict survival according to gene expression profiles. For instance, topology has been employed to handle the high-dimensional data and uncover the shape characteristic of data [31]. Through survival analysis using advanced information technologies for kinds of diseases, potential therapies will be developed and patients may expect better outcome in future.

## Supplementary Material

Figure S1: A rank-2 NMF example in microarray data (see also J. P. Brunet, P. Tamayo, T. R. Golub, and J. P. Mesirov, “Metagenes and molecular pattern discovery using matrix factorization,” PNAS, vol. 101, pp. 4164–4169, 2004.)
Figure S2: Connectivity matrix (*cij*) over 100 runs.
Figure S3: Consensus clustering results of 434 genes without missing values for rank *k* = 2, ... ,5.
Figure S4: The Kaplan-Meier curve results of three kinds of patient division using 434 genes without missing values corresponding to rank *k* = 2, ... ,4.

## Figures and Tables

**Table 1:** 21 important genes selected by lasso regression model.

UNIQID in microarray	Name	Biological function	Correlated cancer or carcinogenesis	coef. in lasso	coef. in elastic net
24432		Unknown		0.1987	0.0864
17316	RPS21	Ribosomal protein	HCC	0.1367	0.0705
15841	MYC	Transcription factor	Many cancers (e.g. DLBCL)	0.0818	0.0713
29250	AARS	tRNA synthase		0.1979	0.0874
30040	PHB2	Mitochondrial morphology	Breast cancer	0.0074	0.0356
30347	SIT1	Lymphoid cell marker		− 0.0440	− 0.0248
19373	HLA-DQA1	MHC class II alpha chain	DLBCL	− 0.1279	− 0.1115
28197	HLA-DPA1	MHC class II alpha chain	DLBCL	− 0.0920	− 0.0799
24396	HLA-DRB1	MHC class II beta chain	DLBCL	− 0.1345	− 0.0877
31957	CD22	B-cell receptor signalling	DLBCL, cancer drug	− 0.0895	− 0.0597
27091	ST6GAL1	Glycosyltransferase	Colorectal cancer	0.1062	0.0741
31316	FCRL3	New CD molecule		0.0324	0.0207
27379	LRMP	Germinal center marker		− 0.0562	− 0.0499
26361		Unknown		− 0.0070	− 0.0037
17723	IGKC	Immunoglobulin light chain		0.0341	0.0360
34407	PTPN6	Protein tyrosine phosphatase	Anaplastic large-cell lymphoma	0.0611	0.0434
24400	MGLL	Monoglyceride lipase		0.0131	0.0216
24395	IFI30	MHC class II Ag processing		− 0.0889	− 0.0635
16972	TXNIP	Interact with thioredoxin	Tumor suppressor gene	0.0659	0.0372
34814	IL23A	Cytokine	Oncogene or tumor suppressor gene	− 0.0114	− 0.0128
17475	HSPA1A	Heat shock protein	Many cancers	− 0.0211	− 0.0166
