# Supplementary material for: Survival Analysis by Penalized Regression and Matrix Factorization
Source: ScientificWorldJournal. 2013 Apr 23;2013:632030. doi: 10.1155/2013/632030 (PMC3655687; doi:10.1155/2013/632030)
Supplement: Supplementary file 1 — Figure S1: A rank-2 NMF example in microarray data (see also J. P. Brunet, P. Tamayo, T. R. Golub, and J. P. Mesirov, “Metagenes and molecular pattern discovery using matrix factorization,” PNAS, vol. 101, pp. 4164–4169, 2004.) Figure S2: Connectivity matrix (cij) over 100 runs. Figure S3: Consensus clustering results of 434 genes without missing values for rank k = 2, ... ,5. Figure S4: The Kaplan-Meier curve results of three kinds of patient division using 434 genes without missing values corresponding to rank k = 2, ... ,4. [file 632030.f1.pdf]

# Supplementary Information on “Survival Analysis by Penalized Regression and Matrix Factorization”

*Yeuntyng Lai, Morihiro Hayashida and Tatsuya Akutsu*

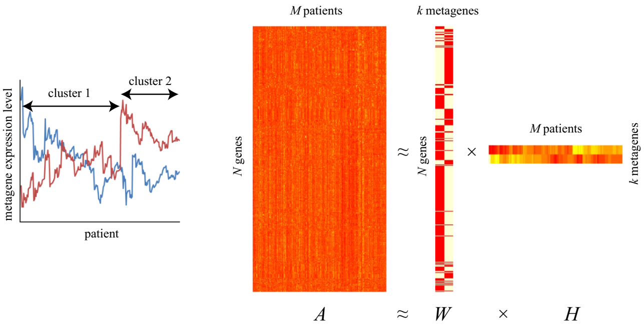


Figure S1: A rank-2 NMF example in microarray data (see also J.P. Brunet, P. Tamayo, T.R. Golub, and J.P. Mesirov, “Metagenes and molecular pattern discovery using matrix factorization,” *PNAS*, vol. 101, pp. 4164–4169, 2004.)

$$
\begin{array}{lcccc}
 & \begin{pmatrix} 1 & 0 & 0 & 1 & \cdots & 1 & 0 \\ \boxed{0} & 1 & & & & & \\ 0 & & 1 & & & & \\ 1 & & & 1 & & & \\ \vdots & & & & \ddots & & \\ 1 & & & & & 1 & \\ 0 & & & & & & 1 \end{pmatrix} & \begin{pmatrix} 1 & 1 & 0 & 0 & \cdots & 1 & 0 \\ \boxed{1} & 1 & & & & & \\ 0 & & 1 & & & & \\ 0 & & & 1 & & & \\ \vdots & & & & \ddots & & \\ 1 & & & & & 1 & \\ 0 & & & & & & 1 \end{pmatrix} & \cdots & \begin{pmatrix} 1 & 1 & 0 & 1 & \cdots & 1 & 0 \\ \boxed{1} & 1 & & & & & \\ 0 & & 1 & & & & \\ 1 & & & 1 & & & \\ \vdots & & & & \ddots & & \\ 1 & & & & & 1 & \\ 0 & & & & & & 1 \end{pmatrix} \\
 & \text{run 1} & \text{run 2} & \cdots & \text{run 100} \\
c_{12} & 0 & 1 & \cdots & 1
\end{array}
$$

Figure S2: Connectivity matrix ($c_{ij}$) over 100 runs.

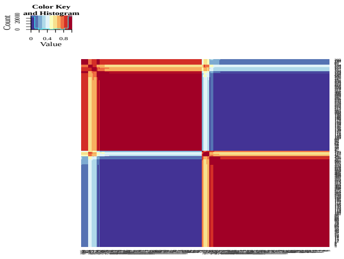


(a) $k = 2$

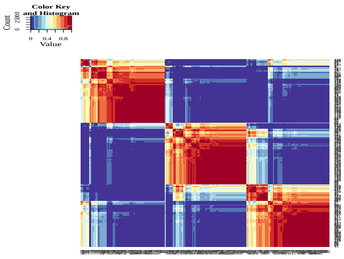


(b) $k = 3$

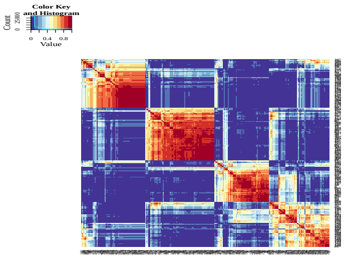


(c) $k = 4$

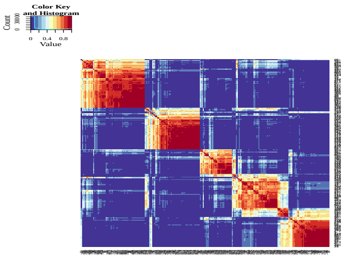


(d) $k = 5$

Figure S3: Consensus clustering results of 434 genes without missing values for rank $k = 2, \ldots, 5$.

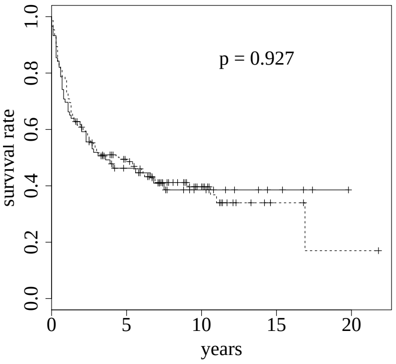


(a) $k = 2$

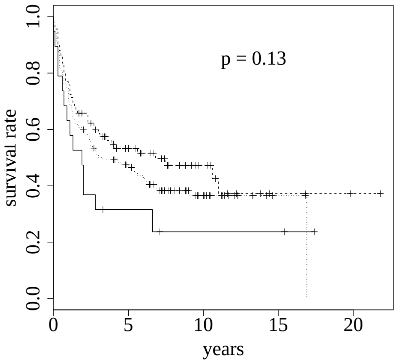


(b) $k = 3$

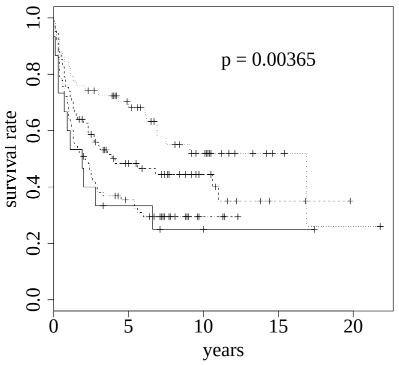


(c) $k = 4$

Figure S4: The Kaplan-Meier curve results of three kinds of patient division using 434 genes without missing values corresponding to rank $k = 2, \ldots, 4$.
